# Normal cohorts in automated brain atrophy estimation: how many healthy subjects to include?

**DOI:** 10.1007/s00330-023-10522-5

**Published:** 2024-01-08

**Authors:** Christian Rubbert, Luisa Wolf, Marius Vach, Vivien L. Ivan, Dennis M. Hedderich, Christian Gaser, Robert Dahnke, Julian Caspers, for the Alzheimer’s Disease Neuroimaging Initiative

**Affiliations:** 1 Department of Diagnostic and Interventional Radiology, Medical Faculty and University Hospital Düsseldorf, Heinrich-Heine-University, Düsseldorf, Germany; 2 Department of Diagnostic and Interventional Neuroradiology, School of Medicine, Technical University of Munich, D-81675 Munich, Germany; 3 Department of Psychiatry and Psychotherapy, Jena University Hospital, D-07745 Jena, Germany; 4 Department of Neurology, Jena University Hospital, D-07745 Jena, Germany; 5 German Center for Mental Health (DZPG), Jena, Germany; 6 Center of Functionally Integrative Neuroscience, Aarhus University, 8000 Aarhus, Denmark

**Keywords:** Atrophy, Brain, Neurodegenerative diseases, Magnetic resonance imaging, Image processing (computer-assisted)

## Abstract

**Objectives:**

This study investigates the influence of normal cohort (NC) size and the impact of different NCs on automated MRI-based brain atrophy estimation.

**Methods:**

A pooled NC of 3945 subjects (NC_pool_) was retrospectively created from five publicly available cohorts. Voxel-wise gray matter volume atrophy maps were calculated for 48 Alzheimer’s disease (AD) patients (55–82 years) using veganbagel and dynamic normal templates with an increasing number of healthy subjects randomly drawn from NC_pool_ (initially three, and finally 100 subjects). Over 100 repeats of the process, the mean over a voxel-wise standard deviation of gray matter *z*-scores was established and plotted against the number of subjects in the templates. The knee point of these curves was defined as the minimum number of subjects required for consistent brain atrophy estimation. Atrophy maps were calculated using each NC for AD patients and matched healthy controls (HC). Two readers rated the extent of mesiotemporal atrophy to discriminate AD/HC.

**Results:**

The maximum knee point was at 15 subjects. For 21 AD/21 HC, a sufficient number of subjects were available in each NC for validation. Readers agreed on the AD diagnosis in all cases (Kappa for the extent of atrophy, 0.98). No differences in diagnoses between NCs were observed (intraclass correlation coefficient, 0.91; Cochran’s *Q*, *p *= 0.19).

**Conclusion:**

At least 15 subjects should be included in age- and sex-specific normal templates for consistent brain atrophy estimation. In the study’s context, qualitative interpretation of regional atrophy allows reliable AD diagnosis with a high inter-reader agreement, irrespective of the NC used.

**Clinical relevance statement:**

The influence of normal cohorts (NCs) on automated brain atrophy estimation, typically comparing individual scans to NCs, remains largely unexplored. Our study establishes the minimum number of NC-subjects needed and demonstrates minimal impact of different NCs on regional atrophy estimation.

**Key Points:**

*• Software-based brain atrophy estimation often relies on normal cohorts for comparisons.*

*• At least 15 subjects must be included in an age- and sex-specific normal cohort.*

*• Using different normal cohorts does not influence regional atrophy estimation.*

## Introduction

Brain atrophy plays a critical role in the progression and diagnosis of various neurodegenerative diseases, such as Alzheimer’s disease (AD) [1], and frontotemporal dementia [2], among others [3–5]. Moreover, brain volume changes are increasingly important for treatment monitoring, such as in multiple sclerosis [6]. However, detecting regional brain volume alterations on MRI, particularly subtle volume losses in the early stages of a disease, can be challenging and is subject to high inter-reader variation [7]. Software-augmented evaluations have demonstrated the potential to reduce this variation [8], which is desirable for accurate diagnosis and treatment monitoring.

Various software approaches are available to aid in detecting and quantifying brain volume changes [9, 10]. Exemplary software includes icobrain dm (icometrix), BIOMETRICA (jung diagnostics), NeuroQuant (Cortechs.ai), Quantib ND (Quantiv), and volBrain (free online tool, https://volbrain.upv.es/ [11]). These tools primarily provide volumes (e.g., in cm^3^) of larger-scale structures, such as the frontal lobe, often in the context of normal percentile curves. Approaches like VEOmorph (VEObrain) [8], VSRAD (Eisai) [12], and veganbagel (Open source, https://github.com/BrainImAccs/veganbagel) [13] derive voxel-wise *z*-score statistics based on (matched, in the case of veganbagel) normal cohorts and offer region-of-interest-based *z*-scores (VEOmorph and VSRAD) or color-coded overlays for interpretation (VEOmorph, VSRAD, and veganbagel). A key difference lies in the interpretability of the results, as color-coded atrophy maps allow for a more refined assessment of atrophy patterns.

One critical aspect of brain atrophy estimation is the use of normal cohorts for comparison. Depending on the approach, a patient may be evaluated in the context of the whole normal cohort, or may be matched to a subset of subjects in the normal cohort, considering factors such as age, sex, and potentially other factors like the scanner model [14–18]. The need for high-quality normal cohorts, ideally well-matched to the local setting, is widely recognized. However, the minimum required number of healthy subjects contributing to a normal cohort for consistent atrophy estimation and the effect of using different normal cohorts on diagnostic reliability have not been well-established in the literature.

Considering these research gaps, this study aims to:

1. determine the minimum number of subjects needed for consistent brain atrophy estimation when using age- and sex-specific normal cohorts, and
2. evaluate the effect of using different normal cohorts on detecting regional atrophy patterns using the mesiotemporal atrophy pattern in AD patients as an example.

By addressing these objectives, our study aims to contribute to a better understanding of the factors influencing the accuracy of automated brain atrophy estimation tools and provide insights into optimizing their use in a clinical setting.

## Methods

The retrospective study has been approved by the local ethics committee (#2021-1424). The need for written informed consent was waived.

### Software for atrophy estimation

**Fig. 1:**
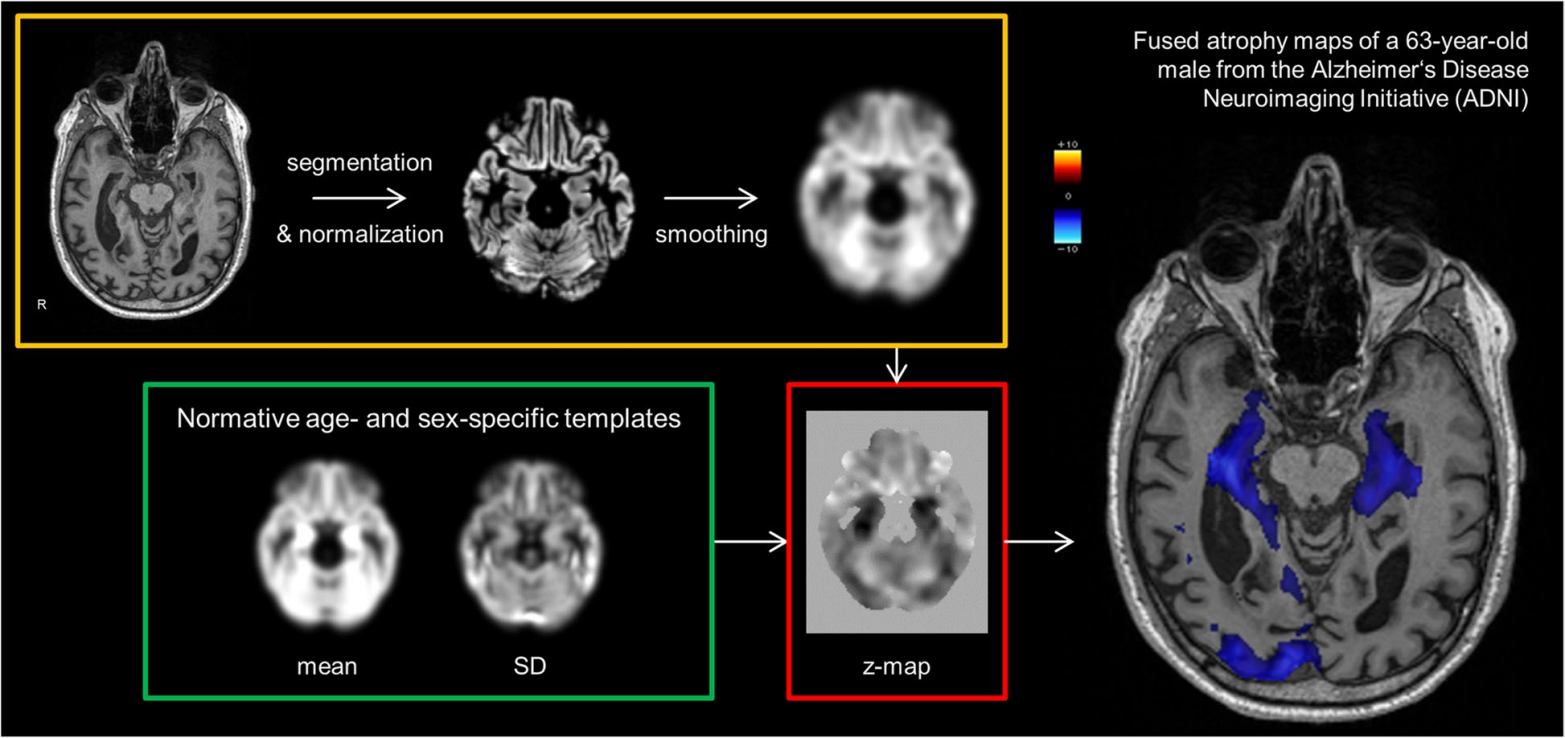
Visualization of the veganbagel workflow. Briefly, standardized preprocessing of structural T1-weighted imaging of subjects from a normal cohort is performed, comprising gray matter normalization, segmentation, modulation, and spatial smoothing using CAT12 for SPM12 with default settings. After preprocessing of healthy subjects, voxel-wise mean and standard deviation (SD) are computed for each sex and age (containing the actual age ±2 years), resulting in age- and sex-specific normal templates (green box). Voxel-wise *z*-score maps (= “atrophy maps”, red box) are then calculated for equally preprocessed subjects (yellow box), which express deviations from the age- and sex-specific normal templates. Atrophy maps may be inversely transformed into subject space and color-coded to generate overlays, with an example from the Alzheimer’s Disease Neuroimaging Initiative (ADNI) for a male aged 63 years of age suffering from Alzheimer’s disease shown on the right

The open-source software veganbagel [13], an automated workflow for generating atrophy maps relative to age- and sex-specific normal templates, was adapted for the analysis. The workflow is depicted in Fig. 1. The Docker-based version of veganbagel was used (https://github.com/BrainImAccs/veganbagel, commit 6a2ac5f), which employs the standalone versions of CAT12.7 (r1713) [19] and SPM12 (version 7771) [20], eliminating the need for a MATLAB-license.

### Consistent brain atrophy estimation

To establish the minimum number of healthy subjects needed for consistent atrophy detection, all healthy subjects from five different public cohorts were included into a pooled normal cohort (NC_pool_), if they met the following criteria: (a) age and sex were known; (b) a structural 3D T1-weighted dataset of the brain with a slice thickness of ≤ 1.5 mm was available; (c) the scan passed the cohort-internal quality control, if applicable; and (d) preprocessing with CAT12 was successful. The normal cohorts comprised the Lifespan Human Connectome Project Aging (HCP-A, started 2009, ongoing [21]), Information eXtraction from Images (IXI, 2005-2006), Nathan Kline Institute–Rockland Sample (Rockland, data sharing started 2010, ongoing [22]) as well as the healthy controls (HC) from the Alzheimer’s Disease Neuroimaging Initiative (ADNI, 2003, ongoing [23]) and the Open Access Series of Imaging Studies 3 (OASIS-3, published 2019, ongoing [24]). The ADNI was launched in 2003 as a public-private partnership, led by Principal Investigator Michael W. Weiner, MD (http://www.adni-info.org/). If there were multiple visits in the study, the first visit was used.

Patients with AD were retrieved from the ADNI database to serve as a surrogate for patients with brain atrophy. Patients with AD were included similar to the healthy subjects, but only if (a) structural imaging with a slice thickness ≤ 1 mm was available, and (b) based on the patients’ sex and age, there were ≥ 100 subjects of the same sex and age ± 2 years available in NC_pool_ (see below).

To establish the minimum number of healthy subjects needed for consistent atrophy estimation, we performed an iterative process on the local High Performance Computing cluster. The process involved repeatedly calculating atrophy maps for each patient with AD using different normal templates, which were dynamically created using an increasingly larger number of randomly selected healthy subjects from NC_pool_. Healthy subjects were selected at random from NC_pool_ to minimize effects of different scanners, sites, and cohorts. Furthermore, the whole process was repeated multiple times. A measure of the variance of the *z*-scores within the atrophy maps is then taken and plotted over the respective number of subjects contributing to the normal templates. We expected to see a considerable variance in *z*-scores with a smaller number of healthy subjects contributing to the normal templates, followed by a steady decrease and, finally, a plateau phase [25].

**Fig. 2:**
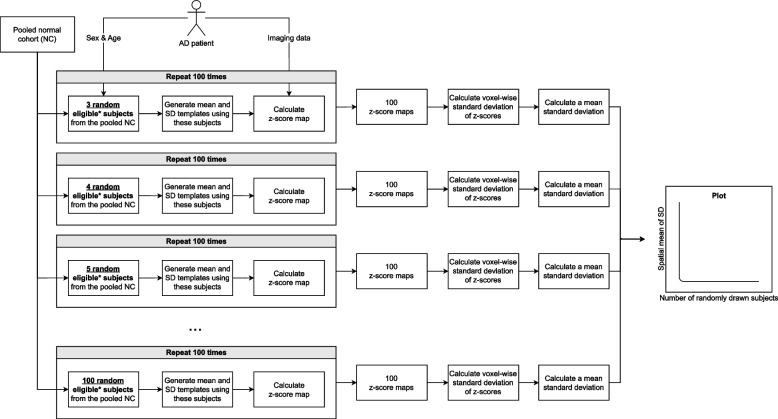
An overview of the process to establish the minimum number of healthy control subjects needed for consistent atrophy estimation. The process was repeated for every patient with AD included in this study. *Healthy subjects of the same sex and ± 2 years of age of the patient with AD were deemed eligible

A detailed overview of the iterative process, which was applied to each patient with AD, can be found in Fig. 2. The process began with the random selection of three age- (± 2 years) and sex-matched healthy subjects from NC_pool_. Following the veganbagel methodology, these subjects were used to create mean and standard deviation (SD) normal templates, which were subsequently employed to compute *z*-score maps for the patient with AD. This entire procedure was carried out 100 times, with each iteration involving the random selection of three new eligible subjects from NC_pool_ to create a fresh normal template. This resulted in 100 distinct *z*-score maps for each AD patient. The voxel-wise SD of *z*-score maps was calculated across the 100 repeats, and in a second step the spatial mean of SD was determined (${\overline{SD} }_{{\text{spatial}}}$), representing the consistency of brain atrophy estimates.

For every AD patient, the process started with three healthy subjects forming the random dynamic normal templates, as described above. This number was incrementally increased by adding one healthy subject at a time (e.g., four subjects drawn, with the procedure above to be repeated 100 times) with up to 100 healthy subjects ultimately contributing to the random dynamic templates. Each subject was included in the normal templates only once, even if multiple scans were available (e.g., due to in-session repeat imaging within ADNI), but may be repetitively included during the 100 repeats. The number of repeats and the upper limit for subjects within the normal templates were informed by results from a prior veganbagel study [13], which utilized templates composed of 10 to 61 subjects. To ensure greater flexibility and comprehensiveness, our study broadened these parameters. The lower limit of three subjects contributing to the normal templates was established, since less than three subjects contributing to the normal templates was determined to yield an unrealistically high variance.

${\overline{SD} }_{{\text{spatial}}}$ was plotted against the number of randomly selected subjects contributing to the normal templates. The “Kneedle” approach was used to determine the knee point of each curve, which involves fitting a smoothing spline to the data, normalizing, and finding the largest distance to a diagonal between the maximum and minimum of the data (https://github.com/etam4260/kneedle.) [26]. The maximum of the knee points across all patients, representing the point of diminishing returns when adding more normal subjects to the normal templates, was defined as the minimum number of subjects required for consistent results in brain atrophy evaluation.

### Effect of different normal cohorts

To test the effect of using different normal cohorts for atrophy estimation on diagnostic reliability, we identified AD patients from the ADNI database for whom the previously established minimum number of age- and sex-specific subjects were available in each of the available non-ADNI normal cohorts (HCP-A, IXI, OASIS-3, and Rockland). HCs from the ADNI database were matched to the patients with AD based on age, sex, and scanner. We generated color-coded atrophy maps for each patient with AD and HC subject using veganbagel, separately using each normal cohort.

The atrophy maps were independently reviewed for the severity of mesiotemporal atrophy in a randomized order by two neuroradiologists with nine years of experience each (C.R. and J.C.), blinded to diagnosis and underlying normal cohort. Mesiotemporal atrophy is both a predictive and prognostic value in AD [27–29]. In the context of the study, it was rated for each hemisphere on a Likert scale, comprised of the following items: 0 = no atrophy, 1 = minimal to moderate atrophy (i.e., a few voxels of atrophy, as indicated by the atrophy map), 2 = marked atrophy (more prominent areas of volume loss, as noted in the atrophy map), 3 = severe atrophy (large areas of volume loss including voxels with *z*-scores ≥ 10). An AD diagnosis was assigned when the bihemispheric score was ≥ 2.

Inter-reader reliability was computed using Cohen’s Kappa, and sensitivity and specificity for the score-based AD diagnosis was determined for each normal cohort. To assess the agreement across the different normal cohorts, a two-way intraclass correlation coefficient was calculated. Cochran’s *Q* test and a pairwise McNemar test with Bonferroni correction were performed to compare the results. *p *< 0.05 was considered statistically significant. Statistical analysis was done using R v4.0.3 [30].

### Data availability

All data used in the manuscript is either publicly available or available to qualified researchers from the respective cohort’s database (see “Acknowledgments”).

## Results

### Consistent brain atrophy estimation

**Fig. 3:**
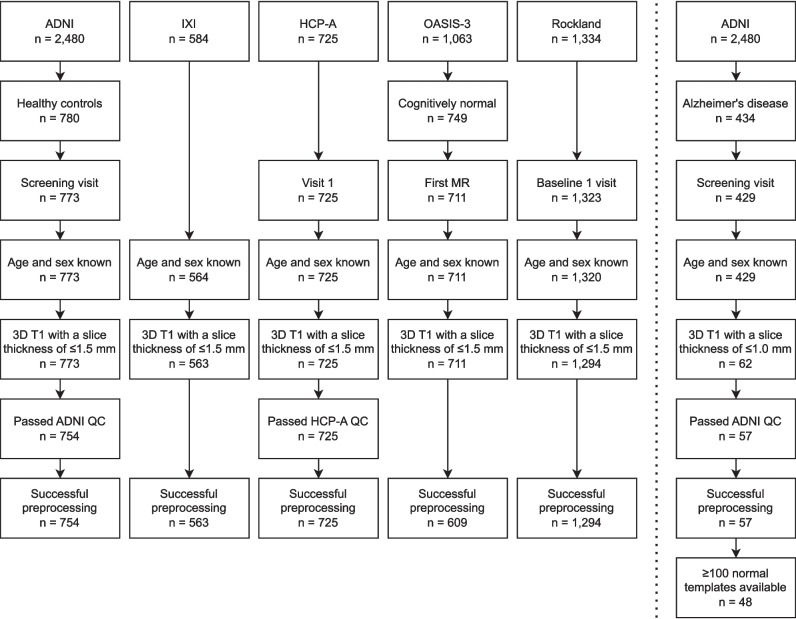
Subjects included into the pooled normal cohort (to the left of the dotted line) and patients with Alzheimer’s disease from the Alzheimer’s Disease Neuroimaging Initiative (ADNI) used for determining the minimum number of subjects to include into a normal cohort (to the right of the dotted line). *From the pooled normal cohort. HCP-A, Lifespan Human Connectome Project Aging; IXI, Information eXtraction from Images; OASIS-3, Open Access Series of Imaging Studies 3; Rockland, Nathan Kline Institute–Rockland Sample; QC, quality control

**Fig. 4:**
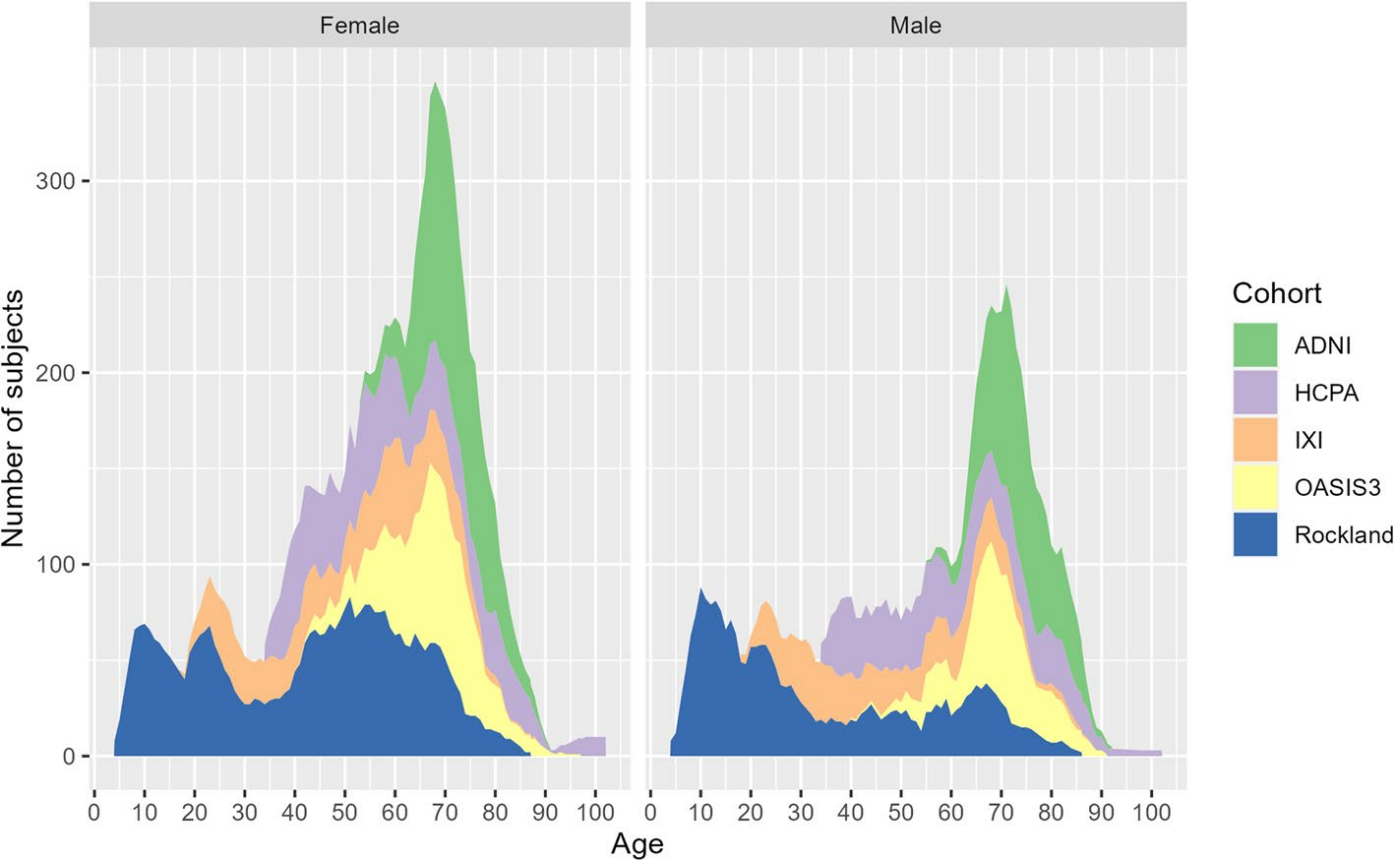
Number of subjects eligible for inclusion in an age- and sex-specific template at each age, shown as a stacked area chart and color-coded by the respective contributing normal cohort. Subjects were considered eligible when they were aged within a range of ± 2 years and were from the same sex

The pooled normal cohort (NC_pool_) consisted of 3945 healthy subjects (55 ± 21 years, 57.9% female, Table 1, Figs. 3 and 4). A total of 48 patients with AD were included in the analysis (73 ± 7 years (range 55–82), 37.5% female, Fig. 3). Thirteen AD patients were scanned using a GE scanner, nine on Philips scanners, and 26 on Siemens scanners. A total of 27 different 3-T scanners were used for the AD patients.

**Table 1:** Descriptive statistics of the normal cohorts and the combined NC_pool_

Cohort	Acronym	Visit	Subjects	Age	T1-weighed images	Scanners^&^	GE/Philips/Siemens (1.5T/3T)
Alzheimer’s Disease Neuroimaging Initiative*	ADNI	Screening	754 (56.4% female)	73 ± 6 (range 55–90)	1049	150	30/17/53% (32/68%)
Lifespan Human Connectome Project Aging	HCP-A	Visit 1	725 (56% female)	60 ± 16 (range 36–100)	725	6	0/0/100% (0/100%)
Information eXtraction from Images	IXI	–	563 (55.6% female)	49 ± 17 (range 19–86)	563	3^$^	12/88/0% (68/32%)
Open Access Series of Imaging Studies 3*	OASIS-3	First MR	609 (58.9% female)	68 ± 9 (range 42–95)	903	5^#^	0/0/100% (3/97%)
Nathan Kline Institute–Rockland Sample	Rockland	Baseline 1	1,294 (60.4% female)	39 ± 22 (range 6–85)	1294	1^+^	0/0/100% (0/100%)
NC_pool_			3945 (57.9% female)	55 ± 21 (range 6–100)	4534	≈165^$#+^	8/15/77% (16/84%)

In the IXI cohort, there was only a single imaging timepoint

GE = General Electric (Boston, MA), Philips = Koninklijke Philips (Amsterdam, Netherlands) and Siemens = Siemens Healthineers (Erlangen, Germany)

^*^From the Alzheimer’s Disease Neuroimaging Initiative and Open Access Series of Imaging Studies 3 cohorts, only the healthy controls are listed

^&^The number of scanners is estimated from the scanner device serial number

^$^Device serial numbers were not available for IXI, number of sites is listed

^#^No device serial number was available for 6 scans

^+^No device serial number was available for 225 scans, but no change of scanner is documented

**Fig. 5:**
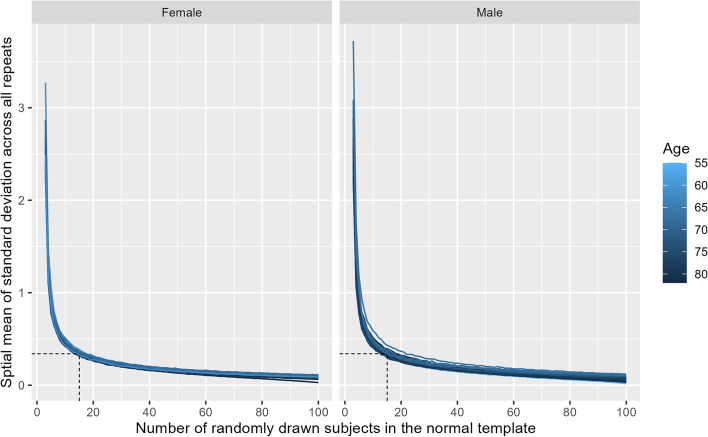
Mean standard deviation of the voxel-wise z-scores over all repeats plotted over the number of randomly drawn subjects included in the dynamically generated normal. The maximum of all established knee points (= 15), representing the point of diminishing returns when adding more normal subjects to the normal templates, is denoted by the dashed black lines. Female patients are shown on the left and male patients on the right

In all AD patients, a sharp drop of the ${\overline{SD} }_{{\text{spatial}}}$ was noted at a small number of healthy subjects included in the normal templates (Fig. 5). The knee points varied across patients, with the smallest at 9 subjects and the largest at 15 subjects (average 11.0 ± 1.2, median 11, inter quartile range 10 to 12). The minimum number of subjects required for consistent results in brain atrophy evaluation was therefore 15, with a corresponding ${\overline{SD} }_{{\text{spatial}}}$ of the *z*-scores of 0.34 ± 0.026 (range 0.297 to 0.432) across all patients.

### Effect of different normal cohorts

**Fig. 6:**
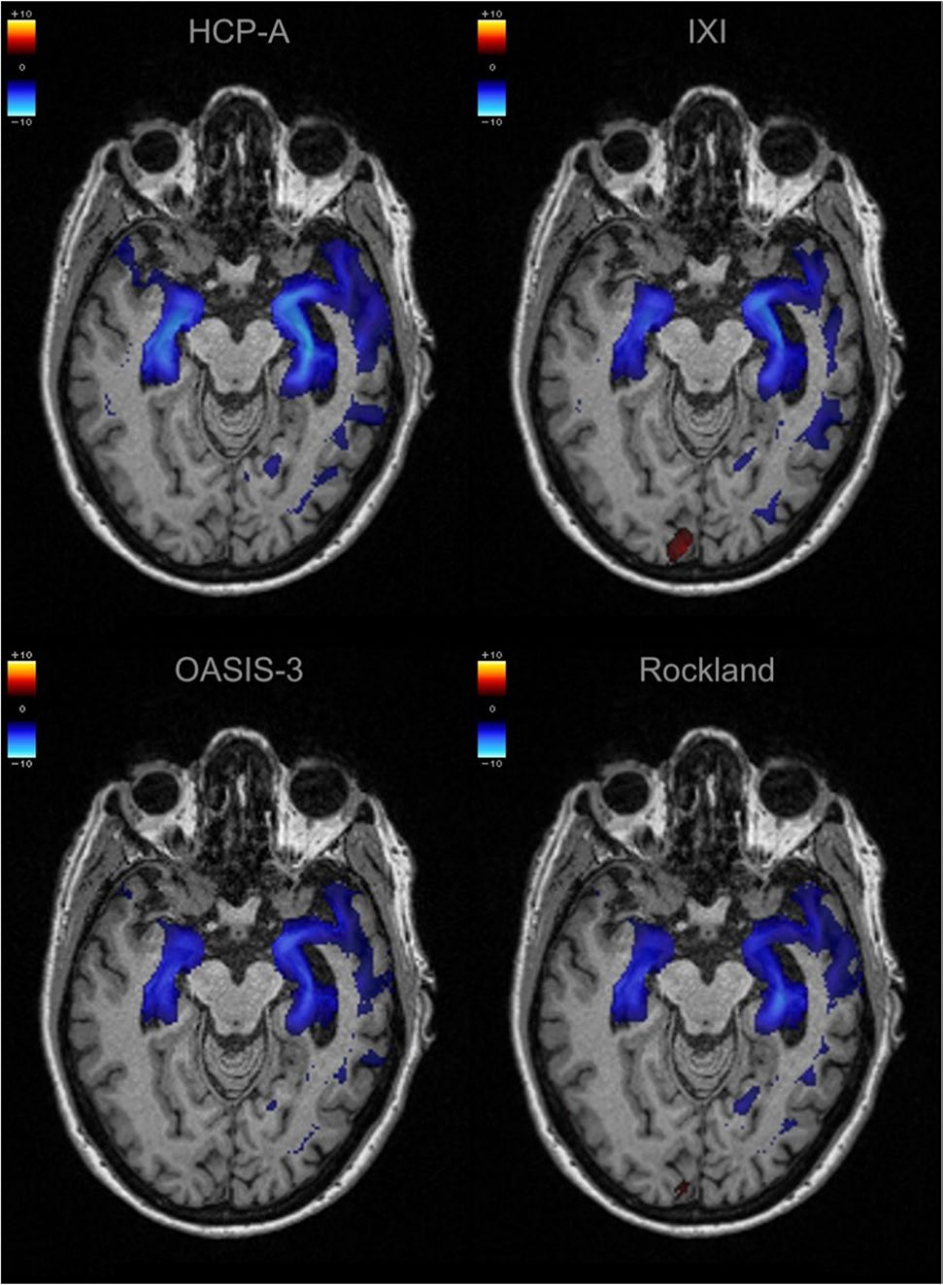
Example of the color-coded z-score maps (= atrophy maps) calculated using veganbagel for a female patient with Alzheimer’s disease (AD), aged 67 years, derived using the four different normal cohorts. The color-coded *z*-score maps are overlaid onto the original 3D T1-weighted MRI acquisitions, shown in the axial plane

A subset of 21 patients with AD and 21 matched HCs had more than 15 healthy subjects available in the HCP-A, IXI, OASIS-3, and Rockland normal cohorts (Table 2). The inter-reader reliability between the two neuroradiologists was high, with an overall Cohen’s Kappa of 0.98 for the extent of the atrophy as determined on the visual rating scale (Table 3). For the individual cohorts, the Cohen’s Kappa was 1 for HCP-A and Rockland, 0.98 for IXI, and 0.93 for OASIS-3. Fig. 6 shows an example of each atrophy map derived.

**Table 2:** Demographic information on the patients with Alzheimer’s disease (AD) and matched healthy control (HC) subjects for testing the effect of using different normal cohorts on regional atrophy detection, as selected from the Alzheimer’s Disease Neuroimaging Initiative (ADNI) database. All scanners were 3T

Group	Subjects	Age	Scanners
Alzheimer’s Disease (AD)	21 (57% female)	68 ± 4 (range 61–74)	GE: 3× DISCOVERY MR750w, 1× DISCOVERY MR750, 1× SIGNA Premier Philips: 2× Achieva, 2× Ingenia, 1× Achieva dStream Siemens: 6× Prisma_fit, 2× Prisma, 1× Skyra, 1× TrioTim, 1× Verio
Matched controls (HC)	21 (57% female)	68 ± 3 (range 63–74)	As above

**Table 3:** Summary of the qualitative ratings on the extent of mesiotemporal atrophy, based on the atrophy maps derived using veganbagel (*HC* healthy control, *AD* Alzheimer’s disease, *R* right, *L* left)

	Reader 1				Reader 2			
	HC		AD		HC		AD	
	R	L	R	L	R	L	R	L
0	83	78	25	18	83	78	24	18
1	1	6	6	7	1	6	7	8
2	–	–	37	42	–	–	37	41
3	–	–	15	17	–	–	15	17

The readers agreed in the diagnosis of AD and HC in all cases. Table 4 lists the respective accuracy, sensitivities, and specificities, as well as the positive and negative predictive value for each reader and normal cohort. The intraclass correlation coefficient across the cohorts was 0.91. Cochran’s *Q* test did not show a significant difference across the different cohorts (*p* = 0.19). Likewise, no significant differences were found in the Bonferroni-corrected pairwise McNemar tests (HCP-A/IXI: *p* = 0.48; HCP-A/OASIS-3: *p* = 0.48; HCP-A/Rockland: *p* = 1; IXI/OASIS-3: *p* = 1; IXI/Rockland: *p* = 0.48; OASIS-3/Rockland: *p* = 0.48).

**Table 4:** Sensitivity and specificity as well as positive and negative predictive value (PPV and NPV) of Alzheimer’s disease vs. healthy control diagnosis based on a scoring of the extent of mesiotemporal atrophy in atrophy maps derived using veganbagel with different normal cohorts

Normal cohort	Accuracy	Sensitivity	Specificity	PPV	NPV
HCP-A	88% (37/42)	76%	100%	100%	81%
IXI	83% (35/42)	71%	95%	94%	77%
OASIS-3	83% (35/42)	67%	100%	100%	75%
Rockland	88% (37/42)	76%	100%	100%	81%

## Discussion

Our study aimed to determine the minimum number of subjects required in a normal cohort for consistent software-based brain atrophy estimation and to evaluate the impact of using different normal cohorts on the qualitative assessment of mesiotemporal atrophy in Alzheimer’s disease. We found that at least 15 healthy subjects should be included in an age- and sex-specific normal cohort for consistent atrophy detection, and that using different normal cohorts does not significantly influence the qualitative evaluation of mesiotemporal atrophy or the imaging-based diagnosis of Alzheimer’s disease.

In our study, we evaluated the open-source software veganbagel [13], which implements an automated assessment of deviation in gray matter volume from a normal cohort using voxel-wise color-coded *z*-score maps. Veganbagel is based on proven methods, namely voxel-based morphometry (VBM) using CAT12 for SPM12. VBM reliably detects patterns in various contexts such as normal aging, neurodegenerative diseases, and psychiatric disorders [15, 31, 32]. More specifically, VBM-based approaches have proven valuable in detecting AD [1] and are routinely used in the clinical diagnosis of AD in Japan [33].

To conduct our analysis with a sufficiently large pool of healthy subjects, we created a pooled normal cohort consisting of subjects from five different normal cohorts with varying objectives, scanners, protocols, and quality. Previous studies have reported differences in quantitative brain atrophy estimation due to factors such as different scanners or protocols [16, 17], while others have demonstrated that volumes of subcortical structures may be interchangeable across different normal cohorts [34]. Given the objective of our analysis, we focused on sex and age as the main influences on brain volume and minimized the potential impact of other factors by not only randomly drawing subjects from the pooled normal cohort, but also by repeating the process 100 times, starting with three and ultimately including up to 100 healthy subjects contributing to the random dynamic normal templates in a computationally intensive approach.

Our study’s results may also enhance radiologists’ comprehension of the mechanisms underpinning automated brain atrophy estimation. Additionally, these findings can guide the decisions-making process when considering commercial solutions, especially in questioning undisclosed, inadequately sized, or insufficiently assessed normal cohorts.

### Minimum number of subjects in a normal cohort

Previous findings in nuclear medicine have suggested a minimum number of 10–20 subjects for a normal cohort when evaluating brain glucose metabolism in the diagnostic workup of dementia [25, 35]. However, the minimum number of subjects in a normal cohort for consistent MRI-based brain atrophy estimation has not been established. Generally, it is assumed that a normal cohort must be as large as possible and as well adapted to a patient as possible with regard to sex, age, scanner, protocol, artifacts, and possibly other factors such as ethnicity or cultural background [14–18, 36].

It is important to recognize that no method for identifying a knee point of a curve is universally accepted, and all approaches rely on approximations dependent on various parameters. A definitive, objective threshold for ${\overline{SD} }_{{\text{spatial}}}$ would be ideal. However, it is important to recognize that any chosen cutoff might possess an element of arbitrariness. In our study, we observed that the variance in *z*-scores sharply diminishes and approaches 0 as more subjects are included in the normal templates. Given that the identified maximum knee point aligns visually with the knee point determined by the Kneedle method, we are confident that, within the scope of the study, a minimum 15 normal subjects is needed for consistent brain atrophy estimation.

### Impact of different normal cohorts on atrophy detection

In the context of our study, we found that qualitative interpretation of regional mesiotemporal atrophy allowed for reliable AD diagnosis when using the different normal cohorts. Our current study outperforms the previously reported sensitivity and specificity for detection of AD in ADNI using veganbagel [13], likely due to evaluating a much smaller sample of patients in the current study. However, the current analysis is focused on the comparison of different normal cohorts, rather than diagnostic accuracy, which allowed for narrower inclusion/exclusion criteria. Nevertheless, the inter-reader agreement for the extent of mesiotemporal atrophy was excellent, which is notable since atrophy assessment on MRI without any software augmentation has been shown to have a low inter-reader agreement [7].

### Limitations and future directions

The limitations of our study include the moderate sample size in the qualitative evaluation of the mesiotemporal atrophy and the evaluation of only one software approach (veganbagel). Other software for brain atrophy estimation, to our knowledge, either is not openly available or does not lend itself to the modifications needed for the conducted analyses. In the case of other open-source alternatives to CAT12/SPM12, such as the FSL or FreeSurfer, no fully integrated software packages for brain atrophy estimation are currently available.

As the number of subjects in the normal templates grows, there is a heighted probability that the same subjects may be repetitively included during the 100 iterations. However, considering the established minimum of 15 subjects and the study’s prerequisite for at least 100 age- and sex-matched subjects for every patient, these overlap likely do not distort our primary conclusions.

The current study leveraged five extensive normal cohorts, enriching the data variety. Yet, these cohorts predominately represent the population of the north-western regions of the world. It is paramount that subsequent studies address the applicability of our findings to the global demographic.

The structure of our experiment, especially its emphasis on numerous iterations, tends to mitigate outlier impacts—whether these outliers arise from atypical, presumed “normal” subjects or from subjects ill-matched to a given patient due to diverse imaging environments. There is an evident need for more focused studies on the resilience of normal templates, particularly those derived from a limited set of subjects.

Future studies should focus on the effects of combining different normal cohorts. A pooled normal cohort for clinical brain atrophy estimation may allow to recruit a sufficient number of healthy subjects for brain atrophy estimation at more extreme ages. Furthermore, a very large and heterogeneous normal cohort would enable more precise matching of patients with regard to factors such as scanners and protocols, enhancing the detection of subtle regional brain atrophy. Last, but not least, patients with other forms of neurodegenerative diseases should be evaluated to ensure that the findings are generalizable across different populations and clinical contexts.

## Conclusion

In summary, our study indicates that a normal cohort should include at least 15 normal subjects, matched for age and sex, to consistently estimate brain atrophy using voxel-based morphometry. In the context of our study, using different normal cohorts did not significantly influence the qualitative assessment of regional mesiotemporal atrophy or the diagnosis of Alzheimer’s disease, and we observed a high inter-reader agreement. It is important to note, however, that these findings are influenced by our study’s particular design and parameters. Thus, caution is necessary when extrapolating these findings to other contexts without fully understanding the inherent assumptions and potential confounding factors.

## Abbreviations

AD: Alzheimer’s disease
ADNI: Alzheimer’s Disease Neuroimaging Initiative
HC: Healthy control
HCP-A: Lifespan Human Connectome Project Aging
IXI: Information eXtraction from Images
NC_pool_: Pooled normal cohort
OASIS-3: Open Access Series of Imaging Studies 3
Rockland: Nathan Kline Institute–Rockland Sample
SD: Standard deviation
VBM: Voxel-based morphometry

## Declarations

### Guarantor

The scientific guarantor of this publication is Christian Rubbert.

### Statistics and biometry

Two of the authors have significant statistical expertise (CR and JC).

### Study subjects or cohorts overlap

Our study is based on five large and well-known public datasets (the Alzheimer’s Disease Neuroimaging Initiative, the Lifespan Human Connectome Project Aging, the Information eXtraction from Images, the Nathan Kline Institute–Rockland Sample, and the Open Access Series of Imaging Studies); therefore, all subjects have extensively reported on before.

### Methodology

• retrospective

• cross-sectional study

• multicenter study
